# Decreased Autonomic Reactivity and Psychiatric Comorbidities in Neurological Patients With Medically Unexplained Sensory Symptoms: A Case-Control Study

**DOI:** 10.3389/fneur.2021.713391

**Published:** 2021-09-07

**Authors:** Victoria Ruschil, Nazar Mazurak, Martin Hofmann, Ekaterina Loskutova, Paul Enck, Tobias Freilinger, Katja Weimer

**Affiliations:** ^1^Department of Neurology and Epileptology, Hertie Institute for Clinical Brain Research, University of Tübingen, Tübingen, Germany; ^2^Department of Psychiatry and Psychotherapy, University of Tübingen, Tübingen, Germany; ^3^Department of Psychosomatic Medicine and Psychotherapy, University Medical Hospital Tübingen, Tübingen, Germany; ^4^Department of Neurology, Klinikum Passau, Passau, Germany; ^5^Department of Psychosomatic Medicine and Psychotherapy, Ulm University Medical Center, Ulm, Germany

**Keywords:** autonomic nervous system, functional disorders, clinical neurophysiology, heart rate variability, medically unexplained sensory symptoms

## Abstract

Up to 48% of patients with medically unexplained symptoms seen in neurological practice suffer from sensory symptoms, which could be of functional nature or secondary to psychiatric disorders. These patients show high medical care utilization causing elevated healthcare costs. Despite the high prevalence, little is known about clinical characteristics and pathophysiological mechanisms. For functional disorders such as irritable bowel syndrome, a reduction of heart rate variability (HRV) has been shown, suggesting a dysfunction of the autonomic nervous system (ANS). The aim of this study was to investigate psychological data and functional changes of the ANS in patients with medically unexplained sensory symptoms (MUSS). In this exploratory pilot study, 16 patients (11 females, 31.6 ± 11.9 years) with MUSS, who were recruited at a single tertiary neurological center, underwent a structured clinical interview (SCID) to evaluate psychiatric comorbidities. Patients and age- and sex-matched healthy volunteers filled in questionnaires, and individual sensory thresholds (perception, pain) were detected by quantitative sensory testing (QST). HRV was assessed at baseline and under three different experimental conditions (tonic pain stimulus, placebo application, cold-face test). All tests were repeated after 6–8 weeks. SCID interviews revealed clinical or subclinical diagnoses of psychiatric comorbidities for 12 patients. Questionnaires assessing somatization, depression, anxiety, and perceived stress significantly discriminated between patients with MUSS and healthy controls. While there was no difference in QST, reduced ANS reactivity was found in patients during experimental conditions, particularly with regard to vagally mediated HRV. Our pilot study of neurological patients with MUSS reveals a high prevalence of psychiatric comorbidities and provides evidence for altered ANS function. Our data thus give insight in possible underlying mechanisms for these symptoms and may open the door for a better diagnostic and therapeutic approach for these patients in the future.

## Introduction

Medically unexplained symptoms (MUS) have a high prevalence of up to 49% in primary medical care (1). Patients with MUS often suffer from the insecurity that an organic origin of their symptoms could be missed and complicate the communication with their treating physicians (2). As a consequence of being more subjectively distressed by their symptoms, patients with MUS have more frequent contacts to physicians, a higher utilization of secondary care, and a higher probability to receive more costly investigations (3–6).

For neurological outpatient clinics in particular, patients with MUS make up a relevant number of about 30% (7). Medically unexplained sensory symptoms (MUSS) are among the most common neurological presentations of MUS next to non-epileptic seizures and functional motor symptoms (8, 9). MUSS can include different sensations such as numbness or paresthesia and either occur in a hemisensory pattern or are distributed unsystematically involving different parts of the body. However, a precise definition of MUS and MUSS is difficult because standardized terms for this heterogenic group of patients are missing. Even by established diagnostic classification systems, e.g., the *International Classification of Diseases, Tenth Revision* (ICD-10) and *Diagnostic and Statistical Manual of Mental Disorders V* (DSM-V), these conditions are not represented sufficiently. On the other hand, the term MUS, which is used to describe symptoms that do not refer to a specific etiology and cannot be explained by another underlying disorder, has also been subject to much criticism for being purely descriptive and negatively defined by the absence of another disorder (10, 11). MUS can include functional disorders that exclusively concentrate on a single organic system (e.g., irritable bowel syndrome) as well as somatoform disorders which are characterized by several persistent and changing symptoms without sufficient organic origin for at least 2 years. At the same time, patients with MUS are often positive for psychiatric diagnoses classified by ICD-10 such as depression or anxiety disorders that only become apparent by elaborate psychological diagnostics and structured clinical interviews (12). The symptomatic overlap of these definitions makes it difficult for treating physicians to differentiate between the terms.

Recently, research started to focus on the biological basis of MUS such as somatoform disorders. So far, neuroendocrinological, immunological, and functional brain imaging findings have been postulated to be involved in the central and peripheral processing of afferent stimuli in patients with MUS (13). Next to environmental factors (14–16), increased sensitization to peripheral stimuli could be at least partially responsible for triggering and maintaining MUS. Another regulatory mechanism for this sensitization could involve the autonomic nervous system (ANS). The link between autonomic regulation and central processing mechanisms has been demonstrated most convincingly for the perception of pain. On the one side, vagal activity has an inhibitory effect on descending pain pathways, whereas sympathetic activity leads to an increased perception of pain (17). On the other side, acute as well as chronic pain is accompanied by elevated sympathetic and reduced parasympathetic activity. Parasympathetic activity correlates with pain experience in daily life as well as with experimentally induced pain in healthy probands (18–20).

For some functional as well as somatoform disorders, such as irritable bowel syndrome and fibromyalgia syndrome, measurable changes in the regulation of the ANS have become apparent (21–23). As the ANS is in permanent control of the heart rhythm, it is responsible for short-term (parasympathetic) or medium-term (baroreflex, sympathetic) changes of beat-to-beat intervals. In comparison with healthy controls, patients with somatoform disorders showed a reduction of heart rate variability (HRV) as a marker for reduced parasympathetic activity. Based on these findings, the reduction of HRV has previously been discussed as a potential biomarker for somatoform as well as functional disorders (24).

Although patients with MUS in general and MUSS in particular form a relevant group of neurological patients, there is still little knowledge on their clinical characteristics and potential physiological biomarkers such as HRV. However, improving the diagnostic approach would be a crucial first step toward a better management of these patients. We hypothesized that patients with MUSS show certain psychological and biophysiological clinical features in comparison with healthy controls. In assumption that MUSS are stress-related disorders, we raised the question if patients with MUSS differed from healthy controls by reduced parasympathetic activity. Therefore, the aim of this explorative pilot study was (1) to examine the eligibility and economic use of psychological questionnaires to detect psychiatric disorders in neurological patients with MUSS and (2) to test HRV as a surrogate marker for ANS function in this specific group. We performed structured clinical interviews, psychological questionnaires, quantitative sensory testing (QST), and HRV analysis in order to generate specific clinical data and to evaluate the regulation of their ANS under different conditions such as pain stimuli, placebo application, and the cold-face test.

## Materials and Methods

### Study Design and Participants

Patients were prospectively recruited from the Department of Neurology of the University Hospital Tübingen, Germany between February 2016 and August 2017. They all received a comprehensive diagnostic work-up during inpatient hospital evaluation. Inclusion criteria were persistent sensory symptoms for a minimum of 4 weeks in patients above 18 years. Underlying organic diseases had been excluded prior to the study according to state-of-the-art neurological standards by experienced neurologists (VS, TF). All patients underwent imaging of the brain and/or parts of the spine. Other diagnostic measures, if considered necessary, included lumbar puncture, evoked potentials, nerve conduction studies, EEG, and sympathetic skin reaction. Exclusion criteria were relevant medical conditions (e.g., diabetes, severe psychiatric disorder, substance-dependence, suicidal ideation, cold urticaria, pregnancy, or breastfeeding) or medication with potential influence on heart rate, peripheral nervous system (PNS), or central nervous system (CNS) (e.g., antiarrhythmic, antiepileptic, antipsychotic, analgesic, etc.) Patients as well as controls were instructed to refrain from smoking for 2 h, from caffeine for 8 h, and from alcohol and any medication for 24 h before testing.

Age- and sex-matched healthy controls were recruited *via* email and advertisements from the catchment area of the University of Tübingen. For practical reasons, we allowed age to differ for ±3 years from the matched patient's age. All participants provided written informed consent before study inclusion. This study was approved by the Ethics Committee of the Medical Faculty of Tübingen University (Project No. 765/2015BO2) and was conducted in accordance with the Declaration of Helsinki.

Patients visited the lab three times for a diagnostic interview (T0) and two experimental sessions (T1, T2). Healthy controls visited the lab at two experimental sessions only (T1, T2). To avoid circadian fluctuations, most T1 and T2 sessions were conducted between 8 a.m. and 2 p.m. and at the same time of the day with small deviations (0 ± 2.5 h) within persons. Controlling for daytime did not affect the results. We planned to investigate all participants again after 6 weeks but had to allow some variance due to time and organizational issues. The time interval between both experimental sessions was 6–8 weeks. Results of T2 are presented in the Supplementary Material.

### Procedures

#### Diagnostic Interview

During the initial visit (T0), patients underwent a structured clinical interview (SCID) according to the *Diagnostic and Statistical Manual of Mental Disorders IV* (DSM-IV) (25) to diagnose mental disorders. All interviews were performed by trained psychologists (KW, EL). Diagnoses were noted when criteria were fully met, and subclinical diagnoses were noted when two criteria at most were not met. Furthermore, patients filled in an adapted version of the German Pain Questionnaire (26) in which “pain” was replaced by “symptoms” to assess location, onset, severity, and course of sensory symptoms.

#### Sensory Threshold and Calibration

**Figure 1:**
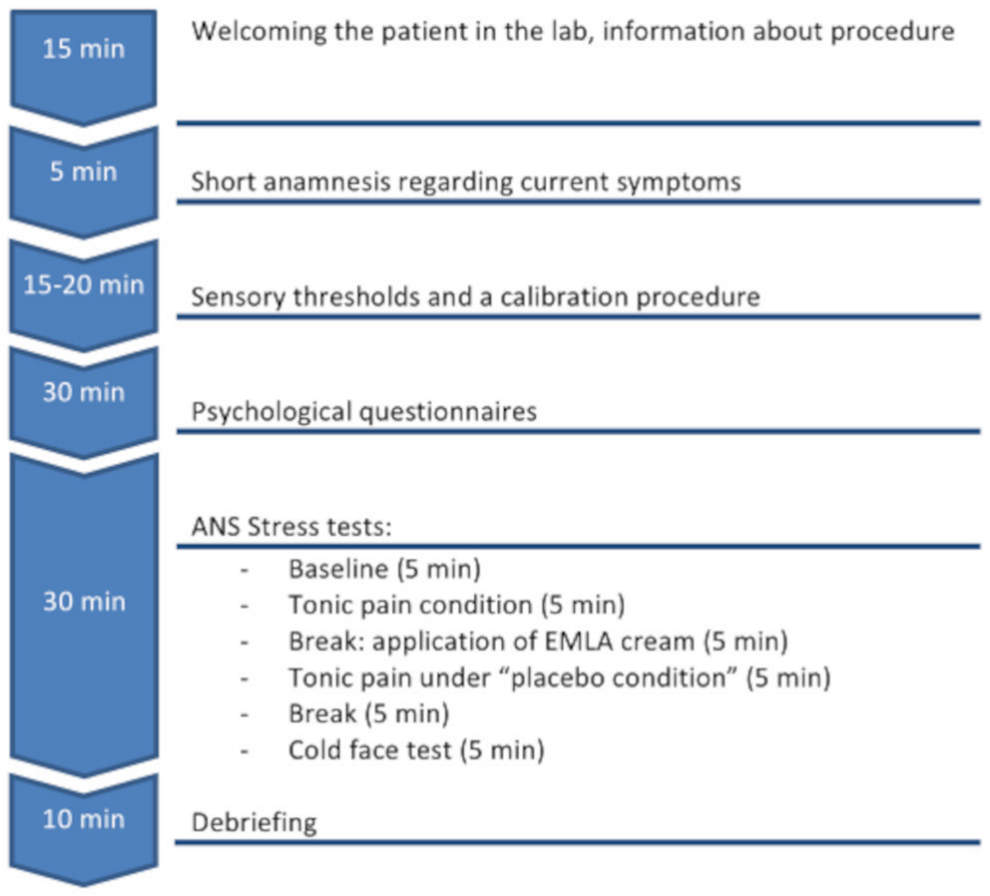
Time lapse of the T1 and T2 experimental sessions.

The two experimental sessions (T1, T2) were identical for all patients and controls (see also flowchart Figure 1).

First, sensory thresholds and a calibration procedure were performed with a Peltier thermode. Three squares of 3 × 3 cm for the positioning of a thermode (Thermal Sensory Analyzer, TSA-II, Medoc Ltd., Israel) were drawn on the volar forearm of the non-dominant hand. The middle square was used for the assessment of the warmth detection threshold (WDT) and the heat pain threshold (HPT) according to the quantitative sensory testing (QST) protocol (27) as well as a calibration procedure. The other two squares were used for a pain and an “analgesic” condition (see below) for which the order of squares was randomized between participants and lab visits. For QST, participants were instructed to press a button as soon as they felt the temperature changes (for WDT) or immediately after the heat stimulus became painful (for HPT). After the button was pressed, the heat sensor returned immediately to the initial temperature of 32°C. The procedure was repeated three times with random breaks (between 3 and 10 s) to avoid learning effects, and means were calculated as outcome measures. During the calibration procedure, six stimuli with a temperature between HPT-1°C and HPT + 1.5°C were applied for 15 s with 10-s breaks in between. The participants were instructed to rate the intensity of pain on a numeric rating scale (NRS) with 0 set for “no pain” and 10 for “the most intensive pain ever.” The temperature that was rated as “4” was used for the further experimental procedure.

#### Psychological Questionnaires

From the Patient Health Questionnaire (PHQ-D) (28), the modules PHQ-15, PHQ-9, “anxiety,” and “stress” were used to assess somatization, depression, anxiety, and psychosocial stress as continuous outcomes (sum scores) as well as binary outcomes for the presence of a somatization disorder, major depression, other depressive syndrome, general anxiety disorder, and panic disorder according to the ICD-10 criteria. The Somatic Symptom Disorder Scale (SSD-12) (29) determined psychological characteristics such as cognitive, affective, and behavioral aspects according to the B criteria of the DSM-V somatic symptom disorder. Sum scores were calculated for all three subscales. The German version of the Perceived Stress Questionnaire-20 (PSQ-20) (30) measured three dimensions of internal stress reactions with the subscales “worries,” “tension,” “joy,” and “demands” as external stressors. Means were calculated for all four subscales and an overall stress mean score.

#### Electrocardiogram and ANS Challenges

Participants were connected to a 3-channel ECG (skin Ag-AgCl electrodes). All data were collected using a TaskForce^®^ Monitor (CNSystems Medizintechnik AG; Graz, Austria). Data were collected continuously during the whole subsequent experimental procedure at a sampling rate of 1,000 Hz. Participants were invited to sit in a comfortable chair and instructed to avoid rapid or intensive movements during the testing procedure. They were asked to sit relaxed for 5 min to collect baseline ECG data.

To evaluate HRV reactivity under different conditions, first a tonic pain stimulus with a heat intensity of “4” on the NRS was used. Stimuli were applied 10 times for a duration of 20 s with 10-s breaks in-between (overall 5 min) to induce a physiological stress response. After each 20 s period, participants were asked to rate the intensity of pain on the same NRS scale as described above.

During the following 5-min break, an application of an inert topical analgesic cream (EMLA cream, AstraZeneca GmbH, Wedel, Germany) was applied to the last marked square on participants' forearm. Participants were told about the nature of EMLA and its ability to modify their perception of heat pain stimuli. However, participants were unaware of the fact that EMLA comes into effect after at least 30 min only (31), to examine a possible placebo effect (32). After 5 min, the skin was thoroughly cleaned and the thermode was put on the pre-treated skin square. The same series of pain stimuli with the assessment of intensity ratings was used as described above.

Finally, after a 5-min break, a cold pack (T ≈ 4°C) was placed on the participants' foreheads for 5 min. This so-called cold-face test (CFT) induces an automatic activation of the parasympathetic nervous system in healthy participants (33).

#### Analysis of Heart Rate Variability

Raw ECG data were exported in the form of the interbeat intervals and stored locally for further processing. Data on autonomic activity were derived from analyses of the HRV based on mathematical transformations of time differences between consecutive heartbeats [interbeat intervals (IBI)]. The detailed background can be found elsewhere [e.g., in (33, 34)]. Analyses were performed with the software Kubios HRV 3.0 (Kubios Oy, Kuopio, Finland) by an experienced researcher (NM). First, 5-min IBI were extracted from the continuous recording for each of the experimental conditions: “baseline,” “tonic pain,” “placebo pain,” and “cold-face test.” IBI were screened for artifacts (by NM) and, if necessary, corrected using the in-built algorithms (threshold-based artifact correction algorithm). Thirty intervals were corrected and six were excluded from further analyses due to insufficient quality of signal. Four of these six intervals were the measures of one control participant (female, 32 years) at T1 and the other two intervals were from one patient (male, 29 years) from both T1 and T2. Consequently, data of those two participants were not considered in repeated measures ANOVAs of HRV. Exclusion of data of those two participants did not influence any other analysis. We evaluated mean IBI in milliseconds as marker of general HRV as well as logarithmized root mean square of successive differences (RMSSD) and logarithmized high-frequency power (HF) as proxies for parasympathetic activity. HF power was obtained using fast Fourier transformation procedure (256 s window width and 50% window overlap).

### Statistical Analysis

Statistical analyses were performed with SPSS Statistics for Windows, Version 25.0 (Armonk, NY: IBM Corp.), and *p*-values of <0.05 were considered statistically significant. Normal distribution of outcome measures was assessed with Shapiro-Wilk tests. Between-group comparisons were performed with Mann-Whitney *U*-tests and with Wilcoxon tests for within-subject comparisons as data were not normally distributed in at least one group. When variances were unequal, adaption of *df* was applied according to Welch and corrected *p*-values are reported only. As HRV parameters (IBI, RMSSD, HF) were normally distributed, they were analyzed with 4 × 2 repeated measures ANOVAs (periods × group) and *t*-tests as *post-hoc* tests. *p*-Values were adjusted according to Greenhouse-Geisser when Mauchly's sphericity test was significant (unadjusted *df* values are reported for reasons of readability).

## Results

**Figure 2:**
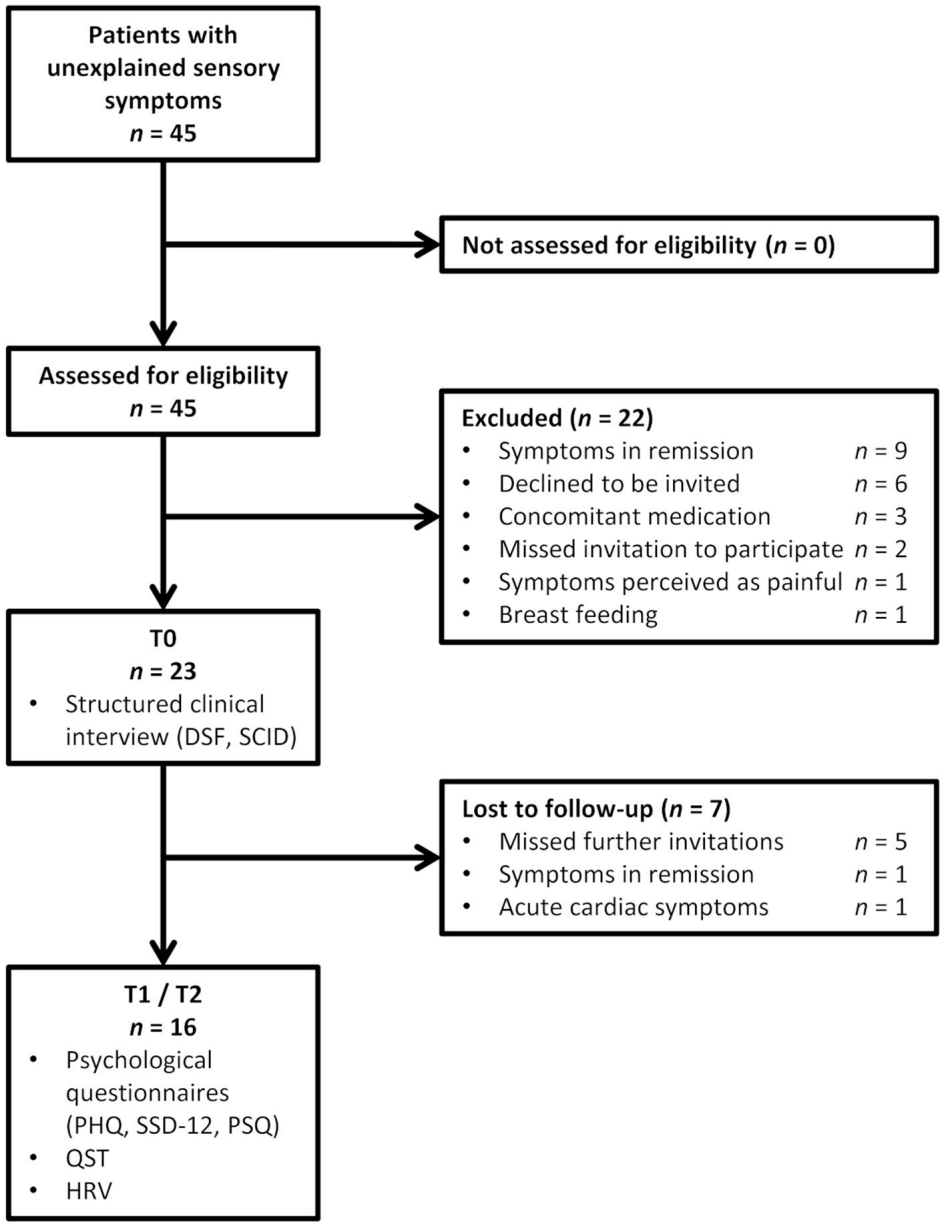
Flowchart for patient inclusion (SCID, Structured clinical interview for mental disorders; DSF, modified German pain questionnaire; PHQ, Patient Health Questionnaire; SSD-12, somatic symptom disorder questionnaire; PSQ, perceived stress questionnaire; QST, quantitative sensory testing; HRV, heart rate variability assessment).

Overall, 45 patients were screened for study inclusion. Twenty-two patients were excluded due to several reasons, and seven patients took part at T0 only as shown in Figure 2.

### Participants' Characteristics and Baseline Data

Sixteen patients, five male and 11 females between 19 and 63 years, completed the study. Time since the onset of sensory symptoms varied between 6 weeks and 36 years and 7 months (Md = 10.25 months). Patients reported to have visited 0 to 18 healthcare professionals (e.g., general practitioners, specialists, psychologists, hospitals, or alternative practitioners) because of the sensory symptoms before contacting our neurological clinic (Table 1). All of them had received (mainly magnetic resonance) imaging of the brain and/or parts of the spine. Further diagnostics included lumbar puncture (*n* = 14), sensory evoked potentials (*n* = 13), nerve conduction studies (*n* = 7), EEG (*n* = 1), and sympathetic skin reaction (*n* = 2). None of the diagnostic interventions had shown abnormal results.

**Table 1:** Demographic and clinical characteristics of patients and controls [data presented as *n* (%), mean (SD), or median (1st quartile−3rd quartile)].

	**Patients** **(*n* = 16)**	**Controls** **(*n* = 16)**
Age (years)	31.63 (11.93)	30.31 (9.39)
**Sex (** ***n*** **, %)**		
- Female	11 (68.8%)	11 (68.8%)
- Male	5 (31.2%)	5 (31.2%)
BMI (kg/m^2^)	24.77 (3.81)	23.57 (2.35)
Age at symptom onset (years)	27.69 (7.76)	
Duration since first symptom (months)	10.25 (3.88–31.50)	
Consulted healthcare practitioners	4.0 (2.0–8.0)	

**Figure 3:**
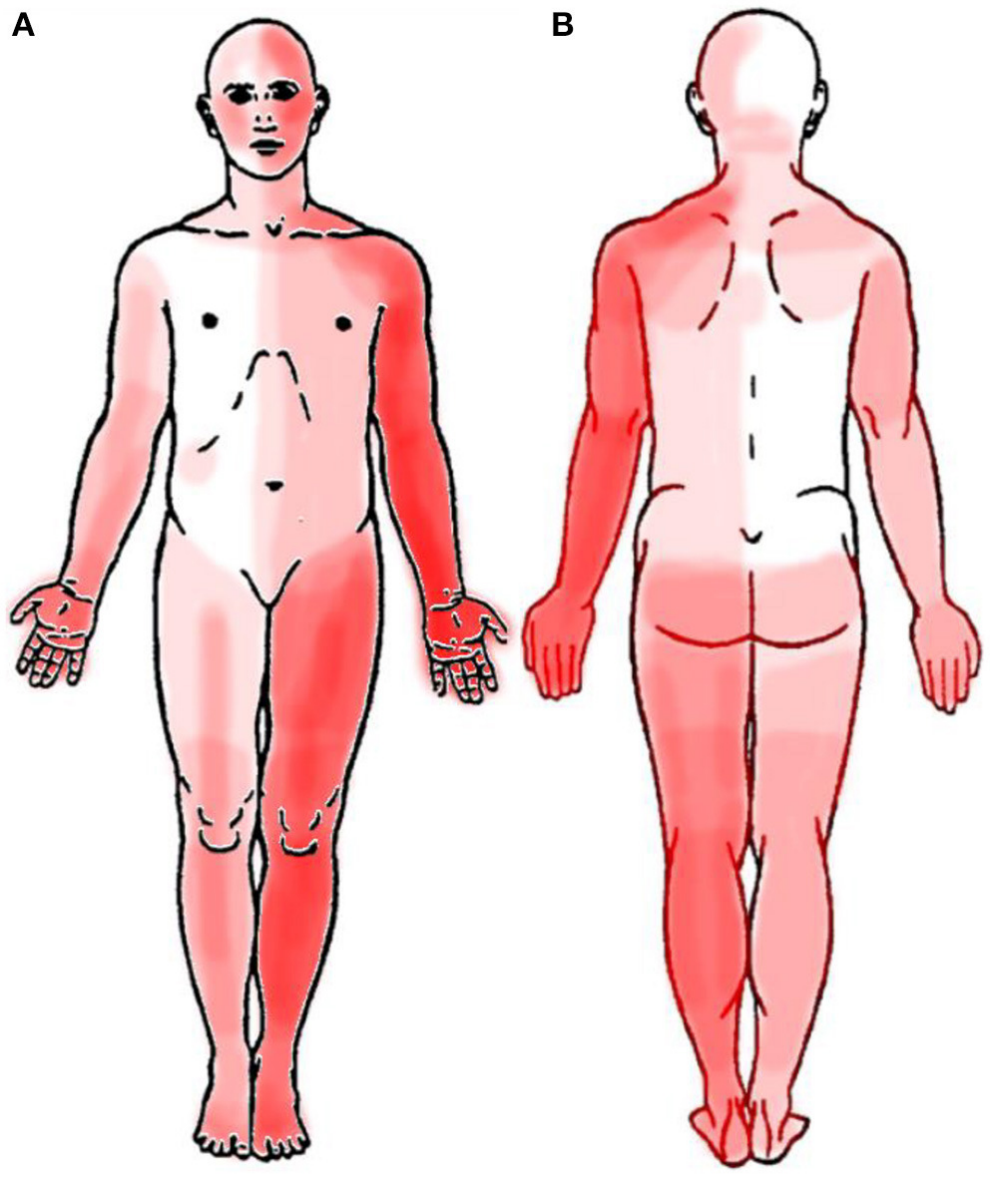
Localization of sensory symptoms in front **(A)** and back **(B)** views in patients [frequency between 0 (white) and 15 (dark red)].

Patients rated the temporal characteristics of their symptoms as follows: 25% described their symptoms as constant with minor fluctuations over time, 12.5% had constant symptoms with strong fluctuations, 31.3% had symptom attacks with symptom-free periods in between, and 25% had constant symptoms with symptom attacks (one patient could not describe the temporal characteristics). The localizations of sensory symptoms were reported differently by each patient; however, a predominance of symptoms was located to the left side of the body (Figure 3).

**Figure 4:**
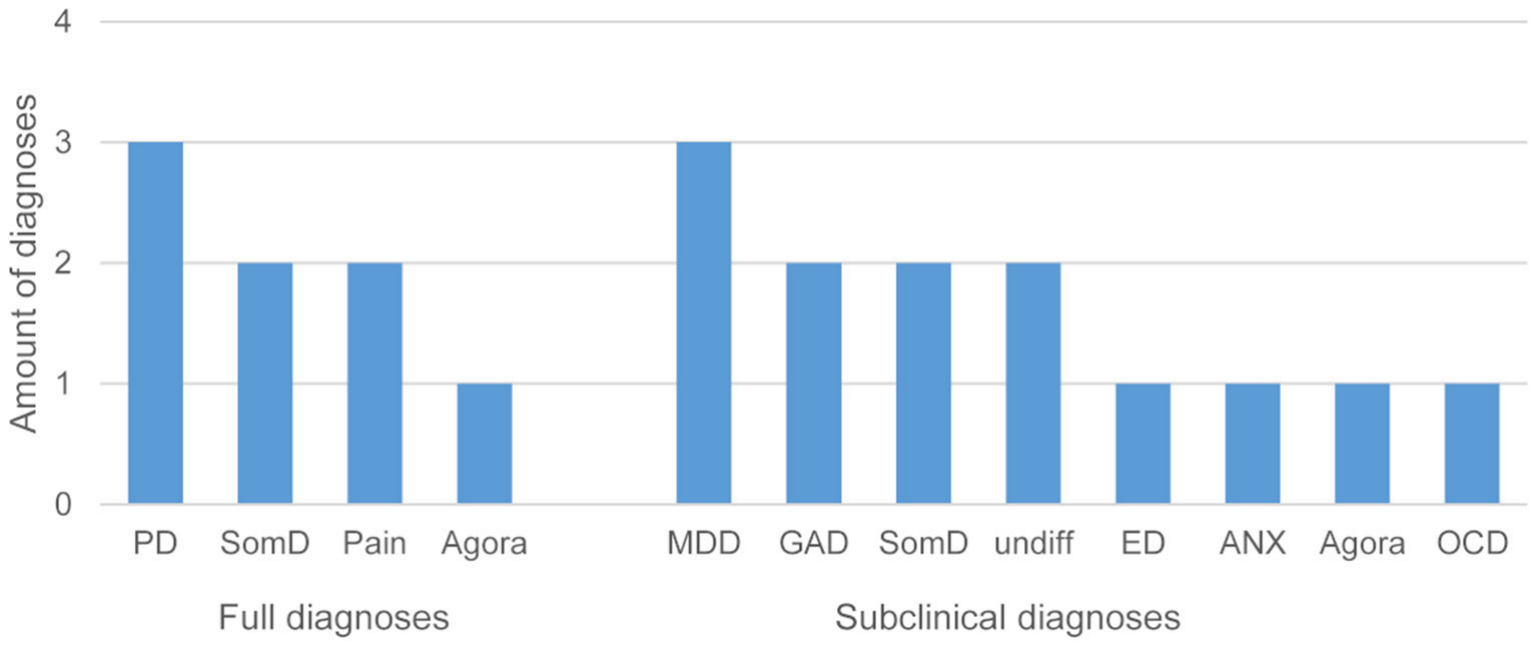
Frequency of fully met and subclinical diagnoses in patients (PD, panic disorder; SomD, somatization disorder; Pain, pain disorder; Agora, agoraphobia without panic disorder; MDD, major depression disorder; GAD, generalized anxiety disorder; undiff, undifferentiated somatoform disorder; ED, eating disorder (binge eating or anorexia nervosa); ANX, anxiety (not otherwise specified); OCD, obsessive-compulsive disorder).

Based on the SCID interviews, 12 patients fulfilled either one or more full clinical or subclinical psychiatric diagnoses defined by the DSM-IV criteria: five patients fulfilled one and two patients fulfilled two diagnoses; four patients met criteria for one subclinical diagnosis, three patients for two, and one patient for four subclinical diagnoses. Next to somatization disorder, pain and panic disorders were represented most prominently (Figure 4). Patients who fulfilled DSM-IV diagnosis were given the advice to consult the Department of Psychosomatic Medicine and Psychotherapy or a local physician/psychotherapist.

### Psychological Questionnaires

A significant discrimination between patients and controls was possible with the scales for somatization, depression, and anxiety of the PHQ and SSD-12 scales. Furthermore, binary outcomes of the PHQ identified patients. The PSQ showed significantly higher values for stress, tension, and worries in patients compared with controls (Table 2).

**Table 2:** Results of psychological questionnaires in patients and controls at T1 [reported as median (1st quartile−3rd quartile)].

	**Patients**	**Controls**	***p*-value (U)**
**Continuous outcomes**			
PHQ somatization	10.50 (5.25–16.00)	3.50 (2.00–5.00)	0.002
PHQ depression	5.00 (1.25–9.50)	2.00 (1.00–2.00)	0.019
PHQ anxiety	4.50 (2.50–7.00)	2.00 (1.00–2.75)	0.007
PHQ stress	4.00 (1.25–6.00)	1.50 (1.00–3.75)	0.056
SSD12 cognitive	5.50 (3.25–8.75)	0.00 (0.00–1.75)	<0.001
SSD12 affective	6.50 (3.00–10.00)	1.00 (0.00–1.00)	<0.001
SSD12 behavioral	3.50 (1.00–7.25)	0.00 (0.00–0.00)	<0.001
PSQ worries	1.90 (1.40–2.80)	1.30 (1.20–1.55)	0.002
PSQ tension	2.40 (1.80–3.10)	1.40 (1.20–1.60)	<0.001
PSQ joy	2.78 (1.80–3.20)	3.50 (3.25–3.80)	<0.001
PSQ demands	2.30 (1.45–2.60)	1.80 (1.45–2.35)	0.324
PSQ sum	2.13 (1.75–2.98)	1.53 (1.31–1.70)	0.001
**Binary outcomes**			
PHQ somatization	7 (43.8%)	0 (0%)	
PHQ major depression	2 (12.5%)	0 (0%)	
PHQ other depression	2 (12.5%)	0 (0%)	
PHQ panic disorder	1 (6.3%)	0 (0%)	
PHQ other anxiety disorder	2 (12.5%)	0 (0%)	

### Quantitative Sensory Testing

The evaluation of the individual WDT and HPT by QST as well as of the applied test temperature according to a VAS rating of 4 showed no difference between patients and healthy controls (Table 3). In addition, no significant difference between patients and controls was found for the intensity of pain based on VAS during tonic pain and after application of a local placebo.

**Table 3:** Warmth detection and heat pain thresholds and pain perception in patients and controls at T1 [reported as median (1st quartile−3rd quartile)].

	**Patients**	**Controls**	***p*-value (U)**
WDT (°C)	33.17 (33.08–33.68)	33.20 (32.89–33.78)	0.637
HPT (°C)	45.63 (43.48–47.82)	45.58 (43.50–46.18)	0.692
Test temperature (according to VAS4) (°C)	45.80 (45.43–46.73)	46.15 (45.13–47.08)	0.763
Tonic pain rating	3.85 (2.23–5.60)	4.75 (4.18–5.38)	0.181
Placebo pain rating	4.60 (3.20–6.60)	4.95 (4.20–5.98)	0.692

### Autonomic Reactivity

**Figure 5:**
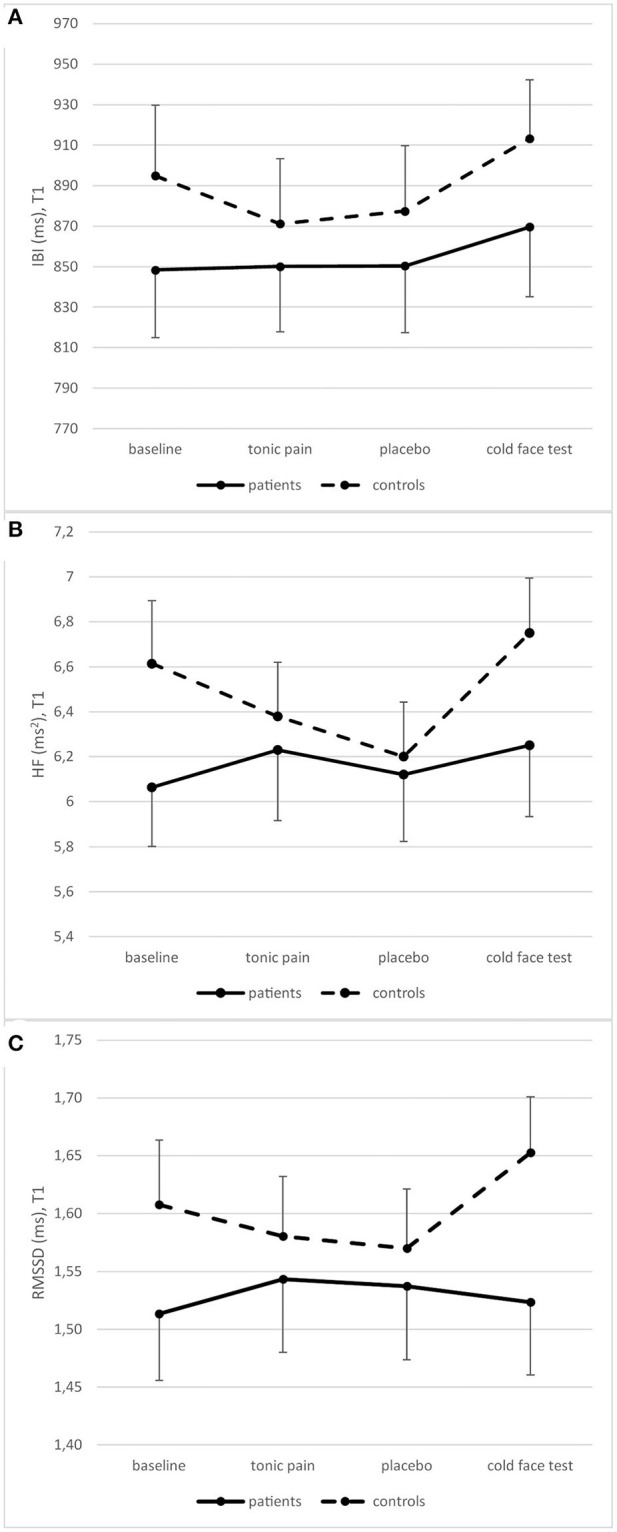
Heart rate variability measured as IBI **(A)**, HF **(B)**, and RMSSD **(C)** in patients and controls at T1 (means and standard errors).

Repeated measures ANOVAs showed a significant change in IBI between recording periods [*F*_(3,84)_ = 8.910, *p* < 0.001], but no difference between groups [*F*_(3,84)_ = 1.790, *p* = 0.170]. There was no change in RMSSD [*F*_(3,84)_ = 2.093, *p* = 0.124], but groups differed significantly [*F*_(3,84)_ = 5.033, *p* = 0.007]. HF significantly changed between periods [*F*_(3,84)_ = 4.441, *p* = 0.013], and this change was different between groups [*F*_(3,84)_ = 3.288, *p* = 0.039] (Figure 5).

*Post-hoc* repeated measures ANOVAs for each group separately showed a significant difference between periods in patients for IBI [*F*_(3,42)_ = 4.405, *p* = 0.009] but not for RMSSD [*F*_(3,42)_ = 0.986, *p* = 0.409] and HF [*F*_(3,42)_ = 1.198, *p* = 0.322]. In contrast, changes over time were significant in all three variables in controls [IBI: *F*_(3,42)_ = 5.697, *p* = 0.010; RMSSD: *F*_(3,42)_ = 5.525, *p* = 0.003; HF: *F*_(3,42)_ = 5.488, *p* = 0.003]. However, there is no significant difference between patients and controls at any single time point (Table 4).

**Table 4:** HRV parameters in patients and controls at T1 (reported as mean ± SD; results of *t*-tests).

	**Patients**	**Controls**	***p*-value**
IBI baseline	848 ± 130	895 ± 135	0.345
IBI pain	850 ± 125	871 ± 124	0.646
IBI placebo	850 ± 128	877 ± 125	0.563
IBI cold-face test	870 ± 134	913 ± 112	0.342
RMSSD baseline	1.51 ± 0.22	1.61 ± 0.22	0.250
RMSSD pain	1.54 ± 0.24	1.58 ± 0.20	0.656
RMSSD placebo	1.54 ± 0.25	1.57 ± 0.20	0.692
RMSSD cold-face test	1.52 ± 0.24	1.65 ± 0.19	0.113
HF baseline	6.06 ± 1.01	6.61 ± 1.08	0.162
HF pain	6.23 ± 1.21	6.38 ± 0.93	0.707
HF placebo	6.12 ± 1.15	6.20 ± 0.94	0.836
HF cold-face test	6.25 ± 1.22	6.75 ± 0.95	0.222

## Discussion

With this explorative pilot study, we could discover measurable deviations of psychophysiological features in a small and well-characterized group of neurological patients with medically unexplained sensory symptoms (MUSS). HRV analysis revealed reduced HRV reactivity pointing at an autonomous dysfunction in 16 patients with MUSS compared with healthy controls (HC). Our findings support a strong psychophysical component of MUSS in line with comparable studies in other somatoform disorders (35). Most patients (12 of 16) fulfilled at least one clinical or subclinical psychiatric diagnosis according to DSM-IV. Among these, somatization, panic, and anxiety disorders were the most common. This finding was supported by further psychological questionnaires (e.g., PHQ and SSD-12) revealing significant differences between patients and controls with regard to somatization, depression, and anxiety. Patients also reported significantly more tension and worries, and less joy. The high prevalence of psychiatric diagnoses in neurological in- and outpatients was comparable with previous studies, especially regarding patients with MUS (12, 36). As the patients scored repeatedly higher in psychological questionnaires at T1 and T2, the data can be considered reliable.

Despite the clinical impression that patients with MUSS are more sensitive for their body functions, there were no differences between patients and healthy controls regarding pain and perception thresholds. This fact was confirmed at T2 where within-subject comparisons showed similar pain and perception thresholds as at T1. However, tonic pain was considered worse in T2 than in T1 by the patients, but not by healthy controls. This could be interpreted as reduced tolerance toward repeated pain stimuli of patients which might be caused by negative expectations. The lack of difference in placebo response following a standard placebo analgesia test (32) supports the concept of normal sensory and pain perception in MUSS as compared with healthy volunteers.

Heart rate responded to different test conditions in both patients and controls; however, vagally mediated HRV did not differ between test conditions in patients on both occasions. It should particularly be mentioned that HRV of patients even did not respond to the cold-face test, a challenge test of the vagus nerve. Thus, patients with MUSS showed a significantly lowered reactivity of the parasympathetic branch of the ANS as compared with healthy controls.

This finding supports the assumption that decreased HRV could work as a surrogate marker for somatoform or “functional disorders” also in these patients as it has previously been discussed for other disorders (21–24). However, a major limitation of our study is the small sample size and a high number of dropouts and exclusions. There was also an imbalance of female to male participants that may weaken the results of the study; however, considering that MUS predominantly occur in female sex, it reflects the normal distribution of these patients (7, 12). The inconsistent level of significance in the evaluation of HRV data could be explained by small group sizes due to the difficult patient collective with many lost in follow-up between T0 and T1. To confirm the effect, studies with larger groups would be necessary.

The results of this pilot study give further insights into the close relationship between MUSS, psychiatric disorders, and the reduction of HRV. As shown in previous studies, depression and anxiety are related to a reduction in HRV independently as well as in combination with physical somatoform symptoms (37). Our results further support the assumption that MUSS add to the group of stress-related disorders involving a complex dysregulation of cognitive appraisal, emotional features, and biophysical symptoms with symptoms interacting and influencing one another.

Regardless of how advanced and subspecialized neurological diagnostics have become during the past decades, patients with different MUS are still a not clearly defined heterogenous group from the neurological as well as a psychosomatic or psychiatric point of view. However, our results indicate that it is possible to further differentiate this group by psychophysiological criteria. Subdividing patients with MUS is of high scientific and clinical relevance and can be a first step toward a better diagnostic access to this difficult group of patients and gives way to potential individualized therapeutic approaches. Psychoeducation and techniques to improve HRV, such as HRV biofeedback (e.g., slow paced breathing), should get more into therapeutic focus for patients with MUS/MUSS in the future (38).

Even though this pilot study might be of little impact considering the small group size, it gives an impetus to consider that neurological patients with MUSS are not only characterized by the absence of another disorder. In our sample, the patients also showed characteristics that could be positively identified by standardized clinical interviews (e.g., depression, anxiety disorders) and physiological measures (reduction of HRV). It is of high practical relevance that treating physicians are sensitized for the psychophysical mechanisms underlying MUS, because it is crucial to identify these patients at an early stage of disease and direct them to a suitable therapeutic intervention in order to prevent chronification of symptoms and unnecessarily high healthcare utilization. To sharpen the view for underlying mechanisms of MUSS and answer the question if HRV analysis would be an eligible clinical tool in the diagnostics of patients with MUSS, larger-scaled studies are necessary.

## Data Availability Statement

The raw data supporting the conclusions of this article will be made available by the authors, without undue reservation.

## Ethics Statement

The studies involving human participants were reviewed and approved by Ethics Committee of the medical faculty of Tübingen University (Project No. 765/2015BO2). The patients/participants provided their written informed consent to participate in this study.

## Author Contributions

TF, PE, and KW contributed to the study design. NM, EL, MH, and KW assessed and analyzed the data. VR and KW did the literature search, created the figures, and wrote the first draft. All authors contributed to the data collection and interpretation of results, reviewed and critically revised the manuscript, and approved the final version for submission.

## Funding

KW received funding from the German Research Foundation for another study (DFG; WE 5658/2-1) and thanks the DFG. VR received funding from the Faculty of Medicine Eberhard Karls University Tübingen for another study (fortüne 2583-0-0).

## Conflict of Interest

VR reports research grants from Novartis, outside the submitted work. TF reports speaker's honoraria from Lilly, from Bayer, from UCB, from Novartis, and from Teva and declares participation in the advisory board of Novartis and Teva outside the submitted work. The remaining authors declare that the research was conducted in the absence of any commercial or financial relationships that could be construed as a potential conflict of interest.

## Publisher's Note

All claims expressed in this article are solely those of the authors and do not necessarily represent those of their affiliated organizations, or those of the publisher, the editors and the reviewers. Any product that may be evaluated in this article, or claim that may be made by its manufacturer, is not guaranteed or endorsed by the publisher.
