# Supplementary material for: Decreased Autonomic Reactivity and Psychiatric Comorbidities in Neurological Patients With Medically Unexplained Sensory Symptoms: A Case-Control Study
Source: Front Neurol. 2021 Sep 7;12:713391. doi: 10.3389/fneur.2021.713391 (PMC8453010; doi:10.3389/fneur.2021.713391)
Supplement: Supplementary file 1 [file Data_Sheet_1.docx]

**Supplemental Material: Data assessed after six to eight weeks (T2)**

After six to eight weeks, all assessments of T1 were repeated with all patients and healthy controls to investigate the retest-reliability of results. Data of T2 and comparisons with data of T1 are presented here.

**Psychological questionnaires**

Compared to controls, patients had higher values in all continuous variables assessed, except in PSQ demands. Furthermore, binary outcomes of the PHQ still identified patients and none of the controls fulfilled criteria of one of the disorders (Table S1).

Table S1. Results of psychological questionnaires in patients and controls at T2 (reported as median [1^st^ quartile – 3^rd^ quartile]).

| **T2** | **Patients** | **Controls** | **p value (U)** |
| --- | --- | --- | --- |
| **continuous outcomes** |  |  |  |
| PHQ somatization | 9.50 [4.25-13.75] | 4.00 [1.50-5.00] | 0.003 |
| PHQ depression | 4.50 [2.25-9.50] | 2.00 [0.25-4.00] | 0.008 |
| PHQ anxiety | 5.50 [3.25-8.00] | 2.50 [1.00-4.00] | 0.005 |
| PHQ stress | 5.00 [2.00-8.00] | 1.00 [0.25-2.00] | 0.009 |
| SSD12 cognitive | 6.00 [4.00-9.75] | 1.50 [0.00-3.00] | 0.001 |
| SSD12 affective | 6.00 [3.25-11.75] | 0.00 [0.00-1.75] | < 0.001 |
| SSD12 behavioral | 3.00 [1.00-7.75] | 0.00 [0.00-1.75] | 0.002 |
| PSQ worries | 2.00 [1.25-2.90] | 1.20 [1.20-1.55] | 0.030 |
| PSQ tension | 2.50 [1.80-3.15] | 1.55 [1.20-1.80] | 0.001 |
| PSQ joy | 2.70 [2.05-3.35] | 3.70 [3.00-3.95] | 0.001 |
| PSQ demands | 2.20 [1.80-2.85] | 1.80 [1.60-2.00] | 0.118 |
| PSQ sum | 2.15 [1.75-2.98] | 1.49 [1.35-1.83] | 0.001 |
| **binary outcomes** |  |  |  |
| PHQ somatization | 6 [37.5%] | 0 [0%] |  |
| PHQ Major Depression | 2 [12.5%] | 0 [0%] |  |
| PHQ other depression | 0 [0%] | 0 [0%] |  |
| PHQ panic disorder | 1 [6.3%] | 0 [0%] |  |
| PHQ other anxiety disorder | 1 [6.3%] | 0 [0%] |  |

Psychological values changed neither in patients nor in healthy controls from T1 to T2 (all p values between 0.164 and 0.875), except a significant increase in SSD12 cognitive (Z = -2.124, p = 0.034) and SSD12 behavioral (Z = -2.232, p = 0.026) in healthy controls. However, values are still significantly lower than in patients.

With regard to somatization, two patients who fulfilled criteria at T1 did not fulfill them at T2 anymore, but another patient fulfilled the criteria at T2. Criteria of Major Depression was fulfilled by two patients on both occasions (one patient still fulfilled criteria and one recovered at T2, but another one fulfilled at T2). The two patients who fulfilled criteria of another depressive disorder were not above the cut-off value at T2 anymore. One patient who fulfilled criteria of another anxiety disorder at T1 recovered, and the same patient still was above the cut-off value of a panic disorder.

**Quantitative sensory testing (QST)**

There were no differences between patients and controls regarding QST outcomes, test temperatures, or pain ratings at T2.

Table S2. Warmth detection and heat pain thresholds, and pain perception in patients and controls at T2 (reported as median [1^st^ quartile – 3^rd^ quartile]).

| **T2** | **Patients** | **Controls** | **p value (U)** |
| --- | --- | --- | --- |
| WDT (°C) | 33.23 [33.05-33.42] | 33.22 [32.84-33.79] | 0.806 |
| HPT (°C) | 46.17 [45.09-47.76] | 46.33 [44.03-47.83] | 0.546 |
| test temperature (according to VAS40) (°C) | 46.40 [45.93-46.90] | 46.25 [45.33-47.50] | 0.720 |
| tonic pain rating | 5.75 [4.75-6.70] | 5.70 [4.43-6.98] | 0.895 |
| placebo pain rating | 5.90 [4.40-7.30] | 5.35 [4.50-5.98] | 0.375 |

Within-subject comparisons showed no difference between T1 and T2 in patients, except for tonic pain with significantly higher ratings at T2 compared to T1 (Z= -3.001, p = 0.003). There were no differences between T1 and T2 in controls in any pain measure.

**Autonomic reactivity**

At T2, 4x2 repeated measures ANOVAs showed that all three HRV parameters changed significantly between periods (IBI: F(3,87) = 9.063, p < 0.001; RMSSD: F(3,87) = 7.727, p < 0.001; HF: F(3,87) = 7.653, p < 0.001). Changes differed between groups regarding RMSSD (F(3,87) = 2.932, p = 0.038), but changes did not differ between groups for IBI (F(3,87) = 2.472, p = 0.067) and HF (F(3,87) = 1.818, p = 0.150) (Fig. S1).

Post-hoc analyses revealed that IBI changed over time in both patients (F(3,42) = 2.845, p = 0.049) and controls (F(3,45) = 7.367, p < 0.001), whereas HF and RMSSD changed over time in controls only (F(3,45) = 6.607, p = 0.001 and F(3,45) = 7.161, p < 0.001, respectively). HF and RMSSD did not change between periods in patients (F(3,42) = 1.834, p = 0.156 and F(3,42) = 1.880, p = 0.168, respectively). However, there is no significant difference between patients and controls at any single time point, except for HF during the cold face test (Table S3).


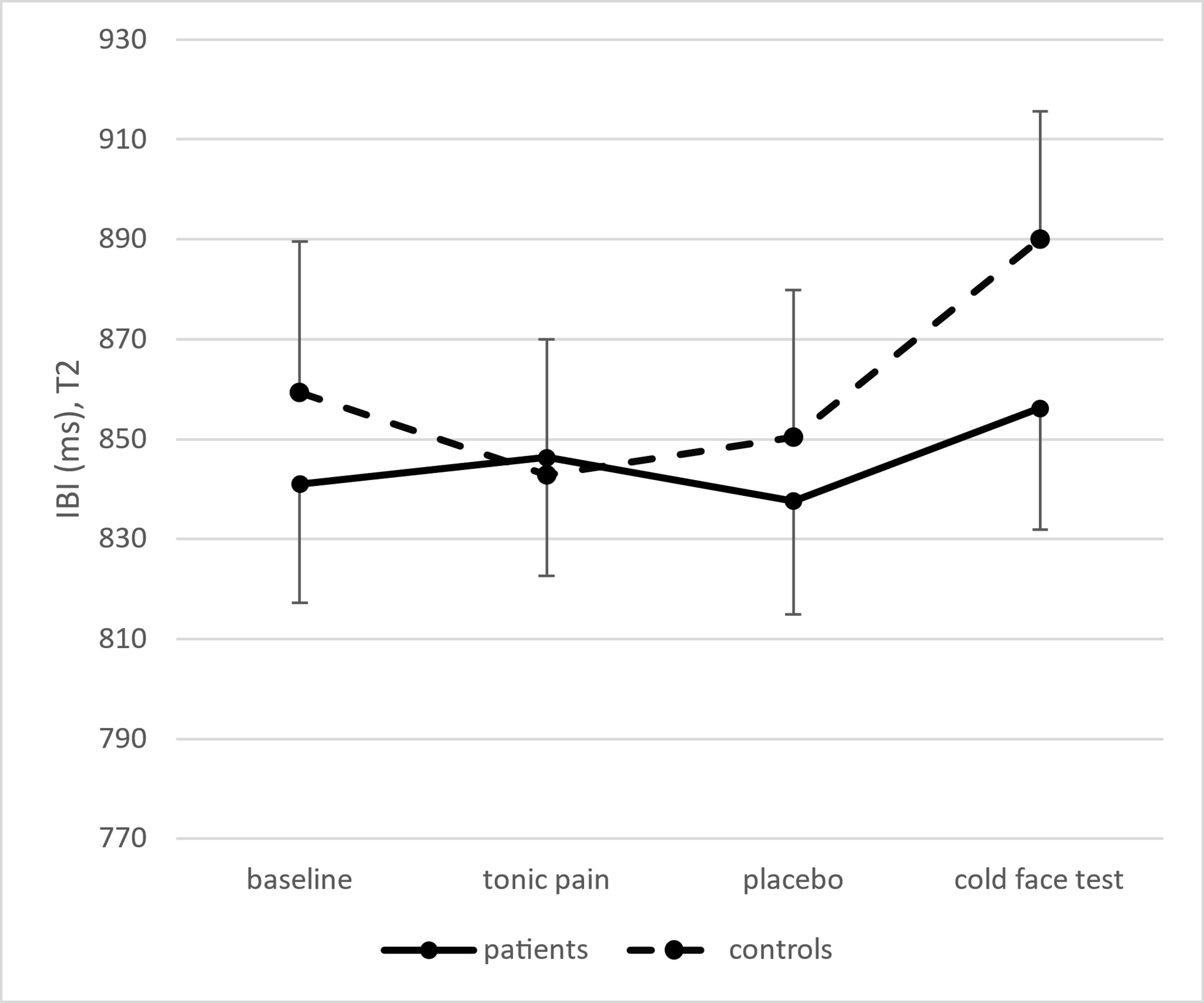

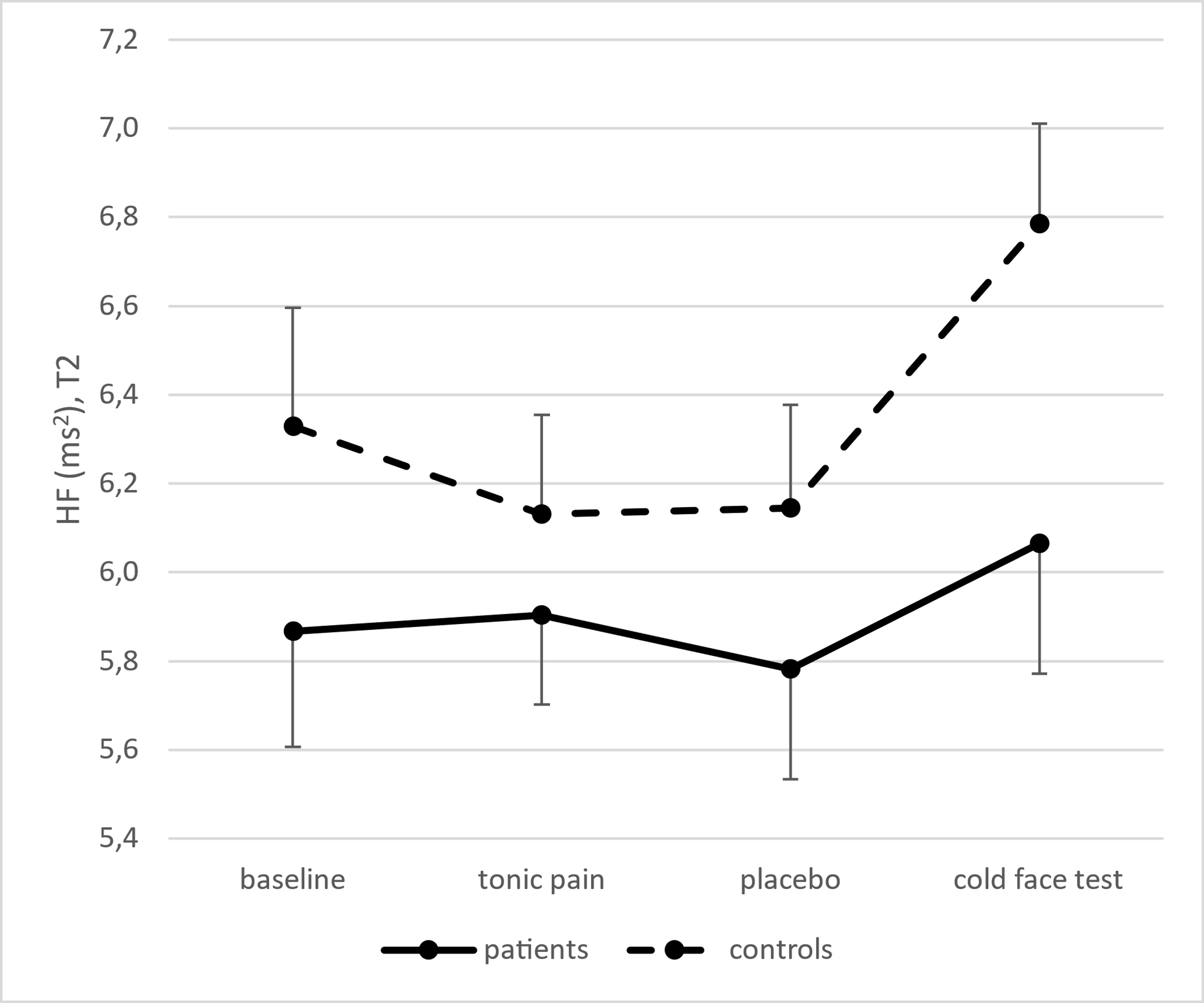


C

B

A


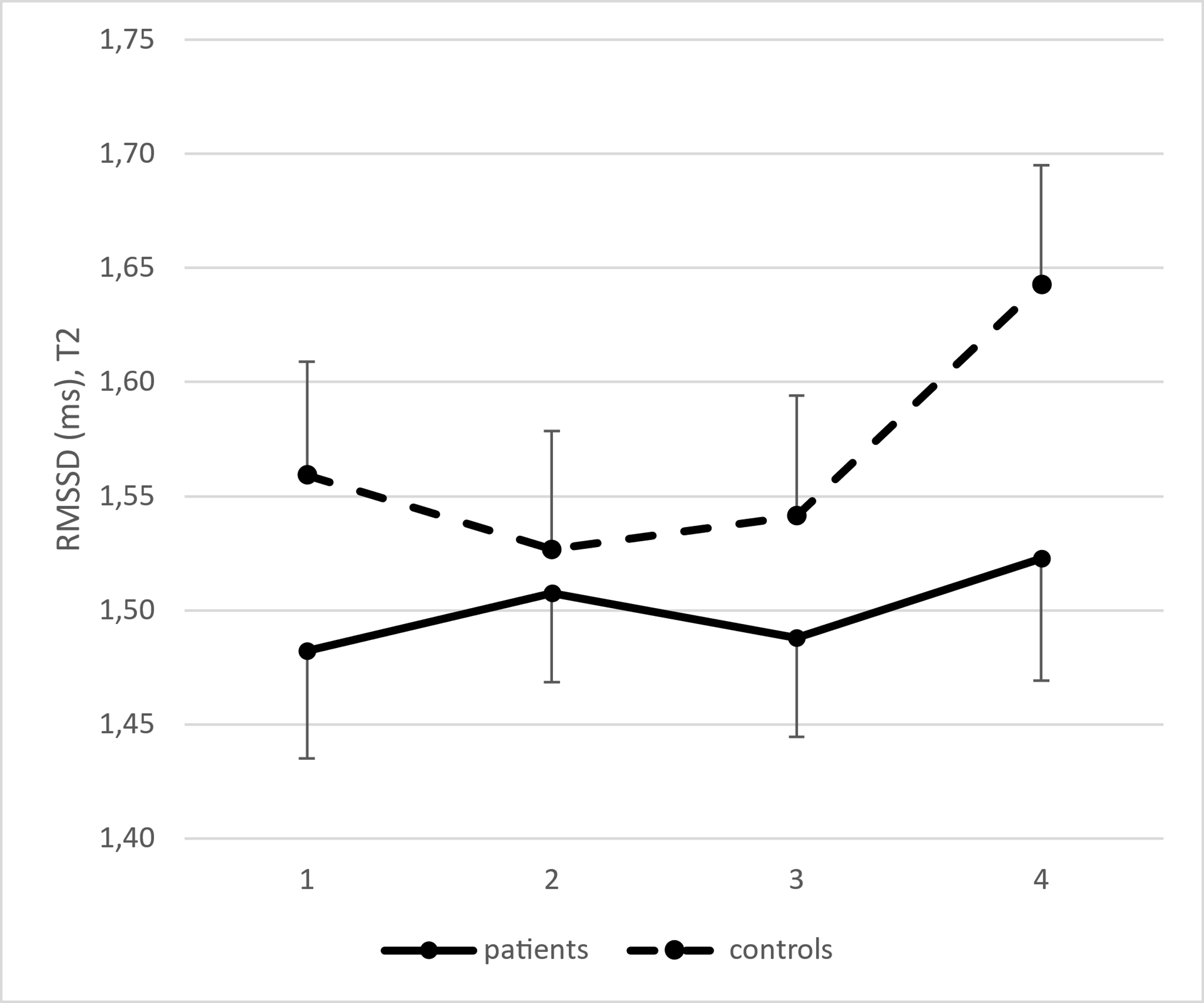


Figure S1: Heart rate variability measured as IBI (A), HF (B), and RMSSD (C) in patients and controls at T2 (means and standard errors).

Table S3. HRV parameters in patients and controls at T2 (reported as mean ± SD; results of t-tests).

|  | **Patients** | **Controls** | **p value** |
| --- | --- | --- | --- |
| IBI baseline | 840 ± 98 | 857 ± 113 | 0.662 |
| IBI pain | 846 ± 98 | 845 ± 102 | 0.976 |
| IBI placebo | 838 ± 88 | 852 ± 110 | 0.689 |
| IBI cold face test | 856 ± 94 | 889 ± 96 | 0.350 |
| RMSSD baseline | 1.48 ± 0.19 | 1.57 ± 0.19 | 0.202 |
| RMSSD pain | 1.50 ± 0.16 | 1.54 ± 0.21 | 0.549 |
| RMSSD placebo | 1.49 ± 0.17 | 1.57 ± 0.22 | 0.274 |
| RMSSD cold face test | 1.52 ± 0.21 | 1.67 ± 0.22 | 0.067 |
| HF baseline | 5.85 ± 1.07 | 6.40 ± 1.03 | 0.157 |
| HF pain | 5.89 ± 0.84 | 6.19 ± 0.88 | 0.334 |
| HF placebo | 5.78 ± 0.96 | 6.23 ± 0.93 | 0.201 |
| HF cold face test | 6.06 ± 1.14 | 6.84 ± 0.88 | 0.042 |

Paired t-tests showed that none of the HRV parameters in patients and controls differed between T1 and T2 (all p values ranged between 0.155 and 0.993).
